# Correlation of left atrial strain with left ventricular end-diastolic pressure in patients with normal left ventricular ejection fraction

**DOI:** 10.1007/s10554-020-01869-7

**Published:** 2020-05-03

**Authors:** Jia-Li Fan, Bo Su, Xin Zhao, Bing-Yuan Zhou, Chang-Sheng Ma, Hai-Peng Wang, Sheng-Da Hu, Ya-Feng Zhou, Yi-Jiao Ju, Ming-Han Wang

**Affiliations:** The First Affiliated Hospital of Soochow University, Suzhou, China

**Keywords:** Left ventricular diastolic dysfunction, Left atrial strain, HFpEF

## Abstract

Left ventricular diastolic dysfunction (LVDD) remains challenging to be assessed by echocardiography. We sought to explore the relationship between left atrial strain and left ventricular (LV) diastolic function in patients with normal left ventricular ejection fraction (LVEF) by invasive left-heart catheterization. 55 consecutive individuals with LVEF > 50% underwent LV catheterization. Standard transthoracic echocardiography was performed during 12 h before or after the procedure. Left atrial (LA) strain were obtained by speckle tracking echocardiography. When LVEF ≥ 50%, the group with elevated left ventricular end-diastolic pressure (LVEDP) (n = 35) showed decreased left atrial reservoir strain (LASr) (35.2 ± 7.7% vs 21.3 ± 7.2%, p < 0.001), left atrial conduit strain (LASct) (17.6 ± 6.3% vs 11.9 ± 4.1%, p < 0.001), left atrial contraction strain (LAScd) (16.6 ± 7.2% vs 9.5 ± 5.0%, p < 0.001) and increased E/e′ ration(8.9 ± 2.6 vs 10.1 ± 3.5, p = 0.17). LVEDP negatively correlated with LASr (R = 0.662, p < 0.001), LASct (R = 0.575, p < 0.001) and LAScd (R = 0.456, p < 0.001), but not with E/e′. LASr, LASct and LAScd were all independent predictors of elevated LVEDP (p < 0.05), with a higher C-statistic for the model including LASr (0.95, 0.86 and 0.93 respectively). The area under the curve (AUC) for LASr is 0.914 (cutoff value is 26.7%, sensitivity is 90%, specificity is 82.9%). In patients with normal LV ejection fraction, left atrial strain presented good correlation with LVEDP, and LASr was superior to LASct and LAScd to predict LVEDP. LA strain demonstrated better agreement with the invasive reference than E/e′.

## Introduction

Left ventricular diastolic dysfunction is an independent predictor of all-cause mortality in the general population, even in the preclinical stage [1], and evidence of LVDD is required for the diagnosis of heart failure with preserved ejection fraction (HFpEF) [2, 3]. So, it is increasingly important to evaluate LVDD accurately in routine clinical practice. Elevated left ventricular (LV) filling pressures are the main physiologic consequence of LV diastolic dysfunction [4]. Although invasive methods are considered the “gold standard’’ for evaluating left ventricular filling pressures and LV diastolic function, echocardiography is routinely used as a noninvasive alternative.

The 2016 ASE/SCAI guidelines streamline the use of four variables into a single algorithm to assess LV diastolic function, while accuracy will be affected in the presence of pulmonary arterial hypertension, severe tricuspid valve lesions, and low right atrial and right ventricular filling pressure etc. [4]. An accurate assessment of left ventricular diastolic function by transthoracic echocardiography is still needed. Speckle tracking is applied to directly reflect the intrinsic deformation of left atrium, which has a relatively independent load environment and geometric model and is less affected by the load change [5, 6]. It is suggested that left atrial strain should be used in diagnosis of LVDD [7]. The previous study indicated that LA strain correlates well with LVEDP, as well as pulmonary capillary wedge pressure [7–10]. While LASct and LAScd are rarely mentioned in previous studies, cut-off value for LA strain is still undefined by invasive gold reference. Our study intended to explore the correlation between LA strain and LV diastolic dysfunction in patients with normal LVEF.

## Methods

### Population

Between June 2018 and November 2019, we prospectively studied 55 patients with LVEF ≥ 50% (mean age, 63 years old, 38 men [69.1%]) referred for left heart catherization, and echocardiography was completed within 12 h before or after catheterization. Patients with confirmed or suspected coronary artery disease underwent left heart catheterization. Those with Atrial fibrillation, ST-segment elevation myocardial infarction (STEMI), non-ST-segment elevation myocardial infarction (NSTEMI), moderate or greater tricuspid regurgitation, moderate or greater mitral regurgitation, any mitral or aortic stenosis, prosthetic valves, hemodynamic instability or poor echocardiography imaging were excluded. The study was approved by the institutional enrolling board of the first affiliated hospital of Soochow university.

### Echocardiography

Echocardiographic measurements were performed by two board-certified echocardiographers blinded to the LVEDP who finalized each measurement by consensus. The two-dimensional echocardiographic imaging of all subjects was performed by GE Vivid E9 and GE Vivid E95 equipment (Norway) 2.5 MHz transducer. The LA dimension was measured in the parasternal long-axis view at the ventricular end-systole. Mitral diastolic inflow was interrogated using pulsed-wave Doppler from the apical four-chamber view at the level of the mitral leaflet tips. Mitral early diastolic peak (E-wave) and late peak (A-wave) velocities and E/A ratio were measured. In the apical two and four-chamber views including the entire left atrium, LAV was determined using biplane Simpson method at end-systolic frames just before mitral valve opening (maximal left atrial volume, LAVmax), at end-diastolic frames coincided with the R-wave on the electrocardiogram (minimal left atrial volume, LAVmin) and during mid-diastole phase which is before electrocardiographic P-wave (left atrial volume before A wave, LAVpre-A). Values were then indexed to the body surface area (LAVimax, LAVimin and LAVIpre-A). left atrial ejection fraction (LAEF) were calculated by LAVImax minus LAVImin. In addition, the septal mitral annulus early (E′) velocity was measured by tissue Doppler imaging, and the E/E′ ratio was calculated using a cutoff value > 15 to represent elevated LV filling pressure. LVEF was measured using Simpson’s method, which was used as a standard index of global LV systolic function. All echocardiographic measurements used in the analysis were averaged from 3 heart beats.

### LA longitudinal strain analysis

**Fig. 1:**
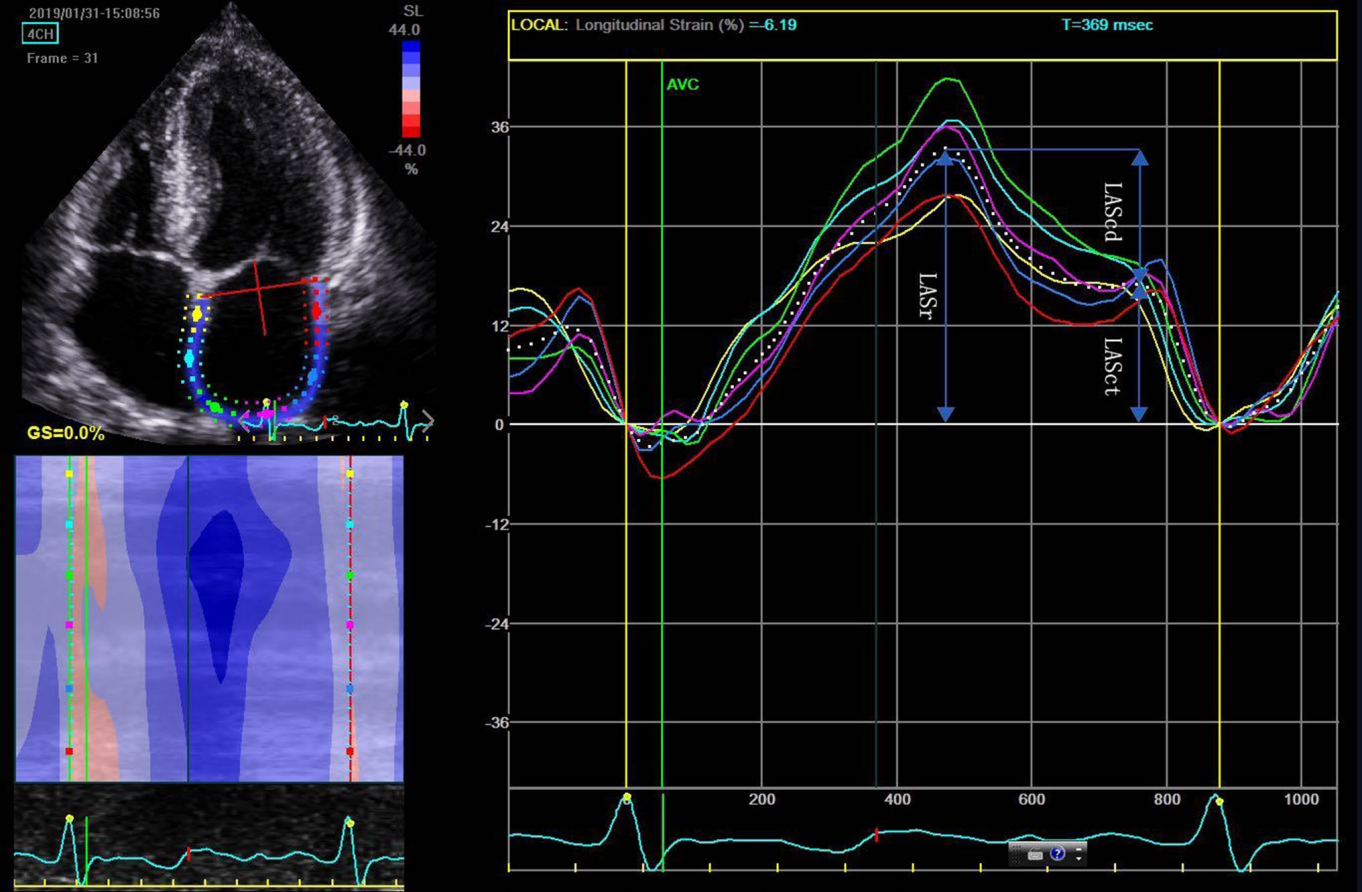
LA two-dimensional strain. Left atrial strain during reservoir phase (LASr) is calculated as difference between onset of filling and end-diastole; Left atrial strain conduit phase (LAScd), calculated as difference between onset of atrial contraction and onset of filling; Left atrial strain contraction phase (LASct), calculated as difference between end-diastole and onset of atrial filling

The two-dimension strain analysis package available on the Echo PAC work station (GE Healthcare) was used to measure the LA strain and strain rate. Similar to the left ventricle, the complete myocardial region of interest (ROI) of the LA is defined by the endocardial border. An adjustable ROI with a default width of 3 mm is recommended. The user can adjust the size and shape to include the thickness of LA wall and to avoid including the pericardium. The software divided the atrial endocardium into six segments, poorly displayed segments were automatically rejected by the software and excluded from the analysis. The global longitudinal strain and strain rate curves were generated by the software for each apical view. The operator could repeat the imaging or change software parameters such as the width of the region of interest and the smoothing functions to obtain satisfactory tracking. Apical four and two-chamber views were obtained in three consecutive heart beats, and electrocardiograph was recorded in the meantime. The LA strain is defined as the absolute strain value in LA three phases, which includes reservoir strain in systole (LASr), conduit strain in early diastole (LASct) and contraction strain in late diastole (LAScd). LV end-diastole was defined as initial zero reference point. The respective strains are LASr, calculated as difference between onset of filling and end-diastole; LAScd, calculated as difference between onset of atrial contraction (before start of Doppler A-wave) and onset of filling; LASct, calculated as difference between end-diastole and onset of atrial filling. The global strain was calculated as the average of both respective values form both views. (Fig. 1).

### Invasive LV pressure measurements

A 6F pigtail catheter was placed in the left ventricle to obtain the invasive LV pressure. Excluding the unstable baseline of LV filling pressure, LVEDP was measured by mean values of 3–5 cardiac cycles. Image J was used to measure LVEDP at the beginning of QRS on Electrocardiography. A LVEDP value > 16 mmHg was defined as elevated LV filling pressure [3, 11]. LVEDP values were measured by two investigators blinded to echocardiographic data.

### Statistical analysis

Analyses were performed using the SPSS 25.0. Continuous variables which are normally distributed are presented as mean ± SD. Variables that are not normally distributed will be presented as median with inter-quartile ranges (IQR = 25th–75th percentile). T test and Mann–Whitney U test was used for comparison of groups. Categorical variables are expressed as absolute numbers and respective percentages, categorical variables were compared with chi squared tests. LA strain and LV diastolic dysfunction parameters such as septal e′ velocity, lateral e′ velocity, E/e′, E/A, LAEF, tricuspid regurgitation velocity, LAVI and LVEDP were evaluated using univariate logistic regression analysis. Differences with p values less than 0.05 were considered significant. The independent predictive value of echocardiographic variables was evaluated in three different multivariate model. Predictors of elevated LVEDP with p value ≤ 0.12 in the univariate analysis and age, were included in multivariate models. In our three models, LASr LASct and LAScd were analyzed separately due to their multicollinearity, with other control variables the same. The C-statistic was calculated in each model, in order to allow comparison between them. Receiver operating characteristics (ROC) curves were created to compare the performance of multiple variables in determining increased LVEDP.

## Results

### Characteristics of population

Patients’ clinical characteristics and echocardiographic measurements are shown in Table 1. LVEDP was elevated in 35 patients (63.6%), and normal in 20 (36.4%) patients. The study population (n = 55) has a mean age of 63 ± 10.0 years, among which 17 (31%) were female. Most characteristics are similar in both groups, such as age, diabetes, CAD etc. Significant differences between the two groups were observed in LA strain (LASr, LASct, LAScd). Vital signs were not significantly different when echocardiography and catheterization were performed.

**Table 1:** Characteristics of patients in two groups defined by LVEDP

Variable	LVEDP ≤ 16 mmHg (n = 20)	LVEDP > 16 mmHg (n = 35)	p
Male	15 (75%)	23 (66%)	0.48
Age	63.8 ± 8	62.4 ± 11.0	0.63
BMI	24.1 ± 3.1	24.5 ± 3	0.83
HR (beats/min)	63.6 ± 10	68.1 ± 9	0.10
Medical history			
HTN	12 (60%)	29 (83%)	0.06
Diabetes	4 (20%)	10 (29%)	0.50
CAD	9 (45%)	11 (31%)	0.32
COPD	1 (5%)	1 (3%)	0.69
CKD(stage ≥ 3)or ESRD	1 (5%)	1 (3%)	0.69
Medications before catheterization			
Diuretic	2	3	1.00
Calcium blocker	5	15	0.19
BB	6	17	0.18
ACEI	5	8	0.86
ARB	2	3	1.00
Angiographic findings			
Stenosis			
None significant stenosis	9	21	0.28
< 50% of diameter	3	5	1.00
> 50% of diameter	6	4	0.18
Total occlusion	2	5	0.97
Numbers of vessels with significant stenosis > 50%			
1	4	6	1.00
2	3	1	0.26
3	1	2	1.00
Vessels with stenosis > 50%			
LM	1	1	1.00
LAD	7	7	0.34
LCX	3	1	0.26
RCA	1	3	1.00
Echocardiographic parameters			
Mitral E (cm/s)	66.1 ± 10.3	68.8 ± 14.7	0.47
Mitral A (cm/s)	76.8 ± 17.8	87.2 ± 21.6	0.07
E/A ratio	0.8 (0.7–0.8)	0.8 (0.6–0.9)	0.19
Septal e′ velocity (cm/s)	6.7 ± 2.0	6.2 ± 1.9	0.356
Lateral e′ velocity (cm/s)	9.4 ± 2.5	8.6 ± 3.1	0.385
E/e' ratio	8.5 (6.9–10.8)	9.9 (8.1–11.1)	0.24
TR peak velocity (m/s)	2.3 (2.1–2.5)	2.3 (2.1–2.6)	0.85
LVEF (%)	66.7 ± 8.9	64.6 ± 7.2	0.95
LAVimax (ml/m^2^)	26.9 ± 9.5	27.7 ± 9.6	0.89
LAVimin (ml/m^2^)	10.6 (7.6–16.9)	10.8 (7.7–13.2)	1.00
LAVi pre-A (ml/m^2^)	17.8 ± 6.8	19.8 ± 6.7	0.54
LAEF (%)	54.5 ± 8.1	55.3 ± 10.3	0.772
LASr (%)	35.2 ± 7.7	21.3 ± 7.2	< 0.001
LASct (%)	17.6 ± 6.3	11.9 ± 4.1	< 0.001
LAScd (%)	14.7 (12.2–19.7)	9.6 (5.8–12.4)	< 0.001

Data are expressed as numbers or as mean ± SD. See text for details

*LVEDP* left ventricular end-diastolic pressure, *HR* heart rate, *CAD* coronary artery disease, *CKD* chronic kidney disease, *COPD* chronic obstructive pulmonary disease, *ESRD* end-stage renal disease, *HTN* hypertension. *BB* β-blocker, *ACEI* angiotensin-converting enzyme, *ARB* angiotensin II receptor blocker, *LM* left main coronary artery, *LAD* left anterior descending coronary artery, *LCX* left circumflex coronary artery, *RCA* right coronary artery, *TR* tricuspid regurgitation, *LVEF* left ventricular ejection fraction, *LAVi* left atrium volume index, *LAEF* left atrial ejection fraction, *LASr* LA reservoir strain, *LASct* LA conduit strain, *LAScd* LA contraction strain

### Correlation between echocardiography assessment and LVEDP

**Fig. 2:**
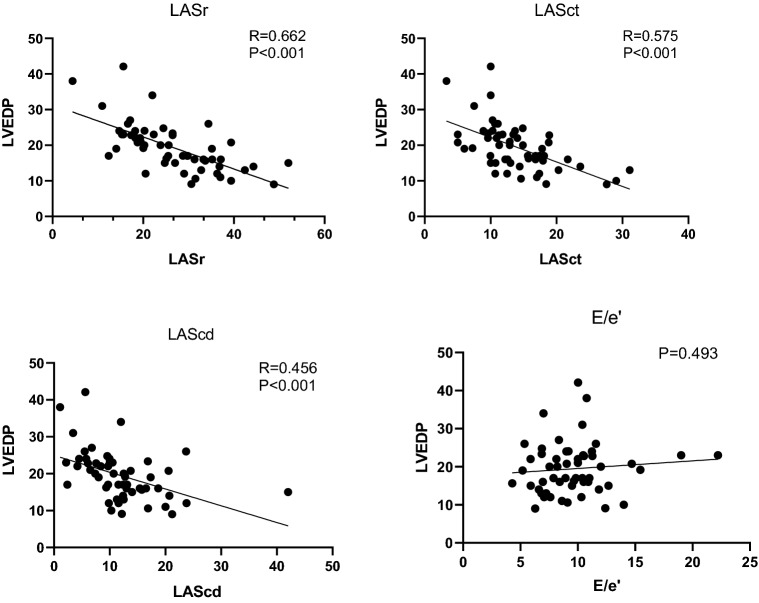
Scatter plots of variables with correlation to LVEDP, respectively LASr, LASct, LAScd and E/e′ ratio

The LA strain and LVEDP are correlated negatively: LASr (p < 0.001, R = 0.662), LASct (p < 0.001, R = 0.575), LAScd (p < 0.001, R = 0.456). No significant correlation was found between the LVEDP and E/e′ (p = 0.493). Figure 2 is scatter diagram of LA strain and E/e′. LASr has a stronger correlation with LVEDP, compared to other variables observed in our study.

The predictive values of LASr, LASct and LAScd were evaluated in three different multivariate logistic regression analyses (Table 2), which contained the same variables (age, Mitral A, HR and hypertension), differing only by control variables. The LASr, LASct and LAScd were independent predictors for LVEDP > 16 mmHg in their respective models. The model shows that LASr has a higher C-statistic when compared to the model with LASct and LAScd.

**Table 2:** Multivariate regression analysis to identify predictors for elevated LVEDP

Model	Variables	Univariate analysis		Multivariate analysis		C-statistic
		OR (95% CI)	p	OR (95% CI)	p	
1	LASr	0.76 (0.67–0.89)	< 0.001	0.75 (0.64–0.88)	0.001	0.95
	Age	0.98 (0.92–1.04)	0.42	0.93 (0.81–1.06)	0.29	
	Mitral A	1.02 (1.00–1.06)	0.12	1.06 (0.99–1.13)	0.12	
	HR	1.07 (1.00–1.14)	0.06	1.04 (0.93–1.17)	0.51	
	HTN	0.36 (0.10–1.27)	0.11	0.22 (0.03–1.73)	0.15	
2	LASct	0.78 (0.65–0.92)	0.003	0.72 (0.57–0.90)	0.004	0.86
	Age	0.98 (0.92–1.04)	0.42	0.95 (0.86–1.04)	0.27	
	Mitral A	1.02 (1.00–1.06)	0.12	1.05 (0.99–1.11)	0.09	
	HR	1.07 (1.00–1.14)	0.06	1.04 (0.95–1.14)	0.42	
	HTN	0.36 (0.10–1.27)	0.11	0.18 (0.04–0.92)	0.04	
3	LAScd	0.76 (0.64–0.90)	0.001	0.71 (0.57–0.89)	0.003	0.93
	Age	0.98 (0.92–1.04)	0.42	0.93 (0.81–1.06)	0.27	
	Mitral A	1.02 (1.00–1.06)	0.12	1.03 (0.98–1.08)	0.21	
	HR	1.07 (1.00–1.14)	0.06	1.08 (0.98–1.20)	0.11	
	HTN	0.36 (0.10–1.27)	0.11	0.39 (0.07–2.12)	0.27	

*CI* confidence interval, *HR* heart rate, *LASr* LA reservoir strain, *LASct* LA conduit strain, *LAScd LA* contraction strain, *HTN* hypertension

### The accuracy of LVEDP > 16 mmHg predicted by LA strain

**Fig. 3:**
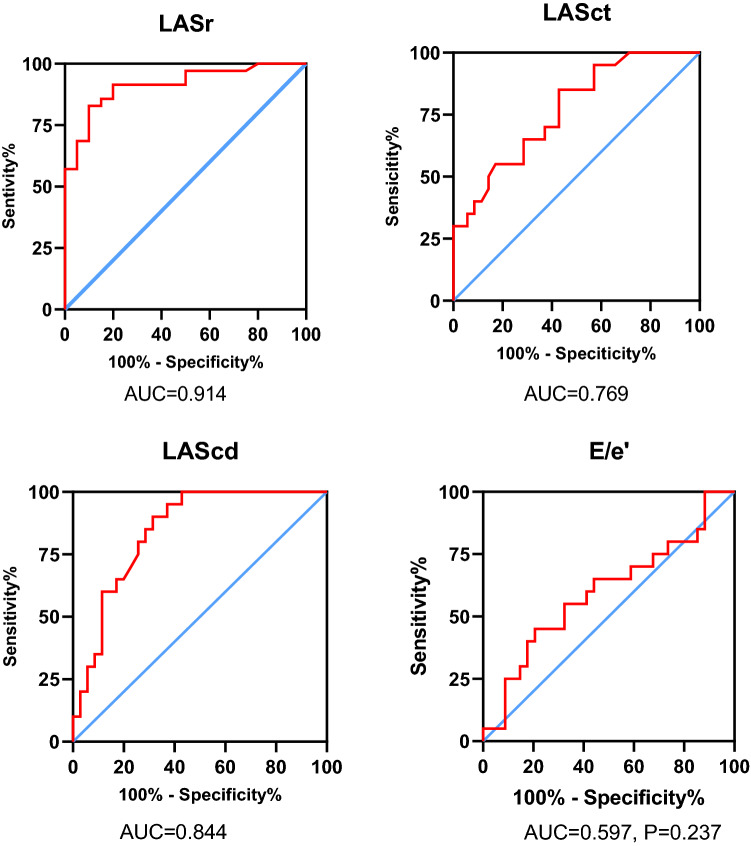
The accuracy of LVEDP > 16 mmHg predicted by LA strain and E/ e′. Receiver operating characteristic (ROC) curves of LASr, LASct and LAScd for prediction of LV filling pressure

Figure 3 shows that LA strain has good diagnostic accuracy for LVEDP > 16 mmHg. The area under the curve (AUC) for LASr is 0.914 (cutoff value is 26.7%, sensitivity is 90%, specificity is 82.9%). For LASct, the AUC is 0.769 (cutoff value is 12.0%, sensitivity is 85%, specificity is 57%). The AUC of LAScd is 0.844 (cutoff value is 11.0%, sensitivity is 90.0%, specificity is 68.6%), which shows that LASr can predict LVEDP better than LASct and LAScd.

## Discussion

Left atrial strain presents significant feasibility and reproducibility, which has recently emerged as a powerful assessment in evaluation of left ventricular diastolic dysfunction [4]. We aim to evaluate the diagnostic accuracy of LA strain for LVDD in patients with normal LVEF by gold standard reference. In this study we demonstrated that left atrial strain was significantly impaired in the group with elevated LVEDP in patients with LVEF ≥ 50%. LASr showed a stronger correlation with LVEDP than with LASct, LAScd and other conventional diastolic echocardiography parameters. LA reservoir strain at cut-off of 26.7% predicted LVEDP > 16 mmHg with 90% sensitivity and 82.9% specificity, which performed a higher diagnosis accuracy than LASct, LAScd and E/e′.

In the early phase of LVDD, reduced ventricular compliance and elevated LVEDP result in decreasing passive early transmitral diastolic flow, then atrial pump function enhances to compensate LV filling. As left ventricular distensibility declines further, atrial pressure rises to maintain cardiac output, which may blunt the compliance of the LA. LA function is impaired as a consequence of chronically elevated LVEDP and decreased LA compliance [12]. This may be the potential mechanism for the inverse correlation between LA strain and LVEDP.

Multiple recent studies have demonstrated the correlation between LA strain and hemodynamic parameters [8, 9], but for patients with LVEF ≥ 50%, investigations with sufficient sample size about correlation between LA strain and LVEDP obtained by invasive gold standard reference, are still needed, and a definitive cut-off value for abnormal LA strain is still undefined.

Our study is consistent with the findings observed by Cameli and colleagues. They found that LASr was strongly negatively correlated with pulmonary capillary wedge pressure (PCWP) in patients with heart failure, which performed excellent sensitivity and specificity of 100% and 93% in assessing PCWP [9]. However, LVEDP > 16 mmHg was defined as LVDD instead of PCWP ≥ 18 mmHg in our study, which may recognize the patients at early stage of LVDD, because elevated PCWP only occurs at a decompensated LA when LVEDP increases significantly. Previous study showed that PCWP frequently underestimates LVEDP, and it only had a moderate ability to discriminate patients with normal or elevated LVEDP [13].

Amita Singh et al. have reported that LASr could predict elevated filling pressure with high accuracy and identified a cutoff value for LASr [8], while we have a lager sample size which may provide a more convincing evidence for cut off value of LA strain in patients with normal LVEF. Besides LASr, our study also demonstrated the correlation between LASct, LAScd and LVEDP. In our study LA strain presented a more sensitive diagnostic value for LVDD than conventional diastolic echocardiography assessments such as E/e′, which indicates that LA strain can recognize patients with LVDD even at early stage.

Poor correlation between E/e′ and LVEDP was observed in this study, which seems to contradict previous reports that E/e′ correlated with PCWP or LVEDP significantly [9, 10, 14, 15]. Possible explanations are as follows. Firstly, Previous study showed that E/e' between 8 and 15 predicts LV Filling Pressure with poor accuracy [16]. However, 34 (61.8%) patients' E/e' ratio is between 8 and 15 in our study. Secondly, the accuracy of E/e′ ratio is uncertain in patients with advance decompensated HF, presence of cardiac resynchronization therapy, mitral annular calcification, surgical rings, prosthetic valves, several mitral regurgitations, atrioventricular or intraventricular conduction delay and symptomatic hypertrophic cardiomyopathy [4, 14, 17].

Our study showed that LAVI correlated with LVEDP poorly. LA dilatation usually implies the chronic, long-term elevation of LA pressure, and LAV can be normal in the early phase of LVDD [18, 19]. Many studies have suggested that LA strain may be superior to LAVi in evaluating LVDD even in the absence of LA enlargement [5, 6], which indicated that LA strain has better sensitivity. Moreover, LAV rarely normalizes with the reduction of LV filling pressure, and strong correlation of reduced LV filling pressure and improved LA strain demonstrates that LA strain may evaluate LA function remodeling better than LA size [20].

Current echocardiographic parameters show moderate sensitivity in evaluating LVDD, with the presence and severity of LVDD failing to be determined for a significant proportion of patients. LA strain measured by speckle tracking echocardiography can reclassify patients even in the early phase of LVDD. LA strain also has advantages when diagnosing LVDD in mitral prosthesis, mitral annular calcification, bundle branch block, post-cardiac surgery and AF. LA strain, with high reproductivity and feasibility, is suggested to be incorporated into routine evaluation of LVDD, especially for patients who are classified in the indeterminate LVDD group using the conventional recommended algorithm.

### Limitations

The sample size in this study is limited, so selection bias still exists, however increasing the number of individuals will reduce the selection bias. The study was conducted on patients from only one medical center, which might impact the population selected and, consequently, the variables analyzed. Left atrium imaging is difficult to obtain and the image definition is poor in some cases, and some echocardiographic images have poor repeatability in left atrial strain.

## Conclusion

For patients with normal LV ejection fraction, left atrial strain presented better correlation with LVEDP than E/e′. Moreover, LASr presented a better diagnostic value in predicting LVEDP than LASct and LAScd. It is suggested that left atrial strain should be used in diagnosis of LVDD.

## References

[1] Redfield MM, Jacobsen SJ, Burnett JC (2003). Burden of systolic and diastolic ventricular dysfunction in the community: appreciating the scope of the heart failure epidemic. JAMA.

[2] Shah KS, Xu H, Matsouaka RA (2017). Heart failure with preserved, borderline, and reduced ejection fraction: 5-year outcomes. J Am Coll Cardiol.

[3] Ponikowski P, Voors AA, Anker SD (2016). 2016 ESC Guidelines for the diagnosis and treatment of acute and chronic heart failure: The Task Force for the diagnosis and treatment of acute and chronic heart failure of the European Society of Cardiology (ESC). Developed with the special contribution of the Heart Failure Association (HFA) of the ESC. Eur J Heart Fail.

[4] Nagueh SF, Smiseth OA, Appleton CP (2016). Recommendations for the evaluation of left ventricular diastolic function by echocardiography: an update from the American Society of Echocardiography and the European Association of Cardiovascular Imaging. J Am Soc Echocardiogr.

[5] Boyd AC, Richards DA, Marwick T (2011). Atrial strain rate is a sensitive measure of alterations in atrial phasic function in healthy ageing. Heart (British Cardiac Society).

[6] Zhang Q, Yip GW, Yu CM (2008). Approaching regional left atrial function by tissue Doppler velocity and strain imaging. Europace.

[7] Thomas L, Marwick TH, Popescu BA (2019). Left atrial structure and function, and left ventricular diastolic dysfunction: JACC state-of-the-art review. J Am Coll Cardiol.

[8] Singh A, Medvedofsky D, Mediratta A (2019). Peak left atrial strain as a single measure for the non-invasive assessment of left ventricular filling pressures. Int J Cardiovasc Imaging.

[9] Cameli M, Lisi M, Mondillo S (2010). Left atrial longitudinal strain by speckle tracking echocardiography correlates well with left ventricular filling pressures in patients with heart failure. Cardiovasc Ultrasound.

[10] Cameli M, Sparla S, Losito M (2016). Correlation of left atrial strain and Doppler measurements with invasive measurement of left ventricular end-diastolic pressure in patients stratified for different values of ejection fraction. Echocardiography.

[11] Kindermann M (2007). How to diagnose diastolic heart failure: a consensus statement on the diagnosis of heart failure with normal left ventricular ejection fraction by the Heart Failure and Echocardiography Associations of the European Society of Cardiology. Eur Heart J.

[12] Dernellis JM, Stefanadis CI, Zacharoulis AA (1998). Left atrial mechanical adaptation to long-standing hemodynamic loads based on pressure-volume relations. Am J Cardiol.

[13] Peverill RE (2015). Left ventricular filling pressure(s): ambiguous and misleading terminology, best abandoned. Int J Cardiol.

[14] Nagueh SF, Smiseth OA, Appleton CP (2016). Recommendations for the evaluation of left ventricular diastolic function by echocardiography: an update from the American Society of Echocardiography and the European Association of Cardiovascular Imaging. Eur Heart J Cardiovasc Imaging.

[15] Kurt M, Tanboga IH, Aksakal E (2012). Relation of left ventricular end-diastolic pressure and N-terminal pro-brain natriuretic peptide level with left atrial deformation parameters. Eur Heart J Cardiovasc Imaging.

[16] Ommen SR, Nishimura RA, Appleton CP (2000). Clinical utility of Doppler echocardiography and tissue Doppler imaging in the estimation of left ventricular filling pressures: a comparative simultaneous Doppler-catheterization study. Circulation.

[17] Mullens W, Borowski AG, Curtin RJ (2009). Tissue Doppler imaging in the estimation of intracardiac filling pressure in decompensated patients with advanced systolic heart failure. Circulation.

[18] Liu YY, Xie MX, Xu JF (2011). Evaluation of left atrial function in patients with coronary artery disease by two-dimensional strain and strain rate imaging. Echocardiography.

[19] Mondillo S, Cameli M, Caputo ML (2011). Early detection of left atrial strain abnormalities by speckle-tracking in hypertensive and diabetic patients with normal left atrial size. J Am Soc Echocardiogr.

[20] Huynh QL, Kalam K, Iannaccone A (2015). Functional and anatomic responses of the left atrium to change in estimated left ventricular filling pressure. J Am Soc Echocardiogr.

